# Development and verification of a nomogram for prediction of recurrence‐free survival in clear cell renal cell carcinoma

**DOI:** 10.1111/jcmm.14748

**Published:** 2019-11-29

**Authors:** Yuanlei Chen, Shangjun Jiang, Zeyi Lu, Dingwei Xue, Liqun Xia, Jieyang Lu, Huan Wang, Liwei Xu, Liyang Li, Gonghui Li

**Affiliations:** ^1^ Department of Urology Sir Run Run Shaw Hospital Zhejiang University School of Medicine Hangzhou China; ^2^ Department of Urology The First People's Hospital of Fuyang Hangzhou China; ^3^ Department of Mathematics and Statistics Science University College of London London UK

**Keywords:** clear cell renal cell carcinoma, nomogram, recurrence‐free survival, risk gene signature

## Abstract

Nowadays, gene expression profiling has been widely used in screening out prognostic biomarkers in numerous kinds of carcinoma. Our studies attempt to construct a clinical nomogram which combines risk gene signature and clinical features for individual recurrent risk assessment and offer personalized managements for clear cell renal cell carcinoma. A total of 580 differentially expressed genes (DEGs) were identified via microarray. Functional analysis revealed that DEGs are of fundamental importance in ccRCC progression and metastasis. In our study, 338 ccRCC patients were retrospectively analysed and a risk gene signature which composed of 5 genes was obtained from a LASSO Cox regression model. Further analysis revealed that identified risk gene signature could usefully distinguish the patients with poor prognosis in training cohort (hazard ratio [HR] = 3.554, 95% confidence interval [CI] 2.261‐7.472, *P* < .0001, n = 107). Moreover, the prognostic value of this gene‐signature was independent of clinical features (*P* = .002). The efficacy of risk gene signature was verified in both internal and external cohorts. The area under receiver operating characteristic curve of this signature was 0.770, 0.765 and 0.774 in the training, testing and external validation cohorts, respectively. Finally, a nomogram was developed for clinicians and did well in the calibration plots. This nomogram based on risk gene signature and clinical features might provide a practical way for recurrence prediction and facilitating personalized managements of ccRCC patients after surgery.

## 1 INTRODUCTION

Renal cell carcinoma (RCC) is one of the most lethal urological tumours, accounting for 2%‐3% of malignancies in the United States.^1^ There are many kinds of histologic subtypes, among which clear cell RCC (ccRCC) constitutes 70% of RCC.^2^ While nephrectomy is curative method for ccRCC, approximately 30% of patients will relapse during the course of disease.^3^

Currently, the American Joint Committee (AJCC) staging system and the Fuhrman grading system have been universally acknowledged for cancer management clinically.^4^ However, clinicians cannot acquire accurate information to estimate recurrence‐free survival (RFS) or overall survival so that providing personalized treatment for ccRCC patients from the TNM and grade classification. This could be ascribed to the biological heterogeneity of cancer, and therefore, molecular exploration may help clinicians precisely make treatment decisions for ccRCC patients according to risk classification via acquiring biomarkers for prediction of recurrence.^5^ Thus, it is urgent to explore new biomarkers for discriminating high‐risk patients who may be inclined to have a higher probability of recurrence, thus offering personalized cancer treatment after surgery.

Clear cell RCC is a highly heterogeneous disease, resulting from complicated interaction between genetic and environmental factors.^6^ Analysing gene expression profiles of different cancer tissues or cells, with different tumour stages, may be helpful for identification of characteristic risk gene signature in cancer. Nowadays, many researchers have focused on the gene expression profiles of ccRCC and tried to illuminate the underlying mechanism of progression.^7^ However, few of them are used clinically. Therefore, identifying a more precise and practical risk gene model for predicting prognosis is urgently needed.

In the present study, we identified the differentially expressed genes between the normal kidney samples and ccRCC tumour tissues by gene expression microarray. A risk gene signature that can reflect the biological heterogeneity of different ccRCC patients and effectively predict clinical RFS was established via integrating gene expression profiles with matched clinical patient information. Moreover, we combined both genomic and clinical features of patients to construct a nomogram model for more accurate recurrence evaluation and facilitating personalized management of ccRCC patients after surgery.

## 2 MATERIALS AND METHODS

### 2.1 Study design and ccRCC specimen cohorts

**Figure 1:**
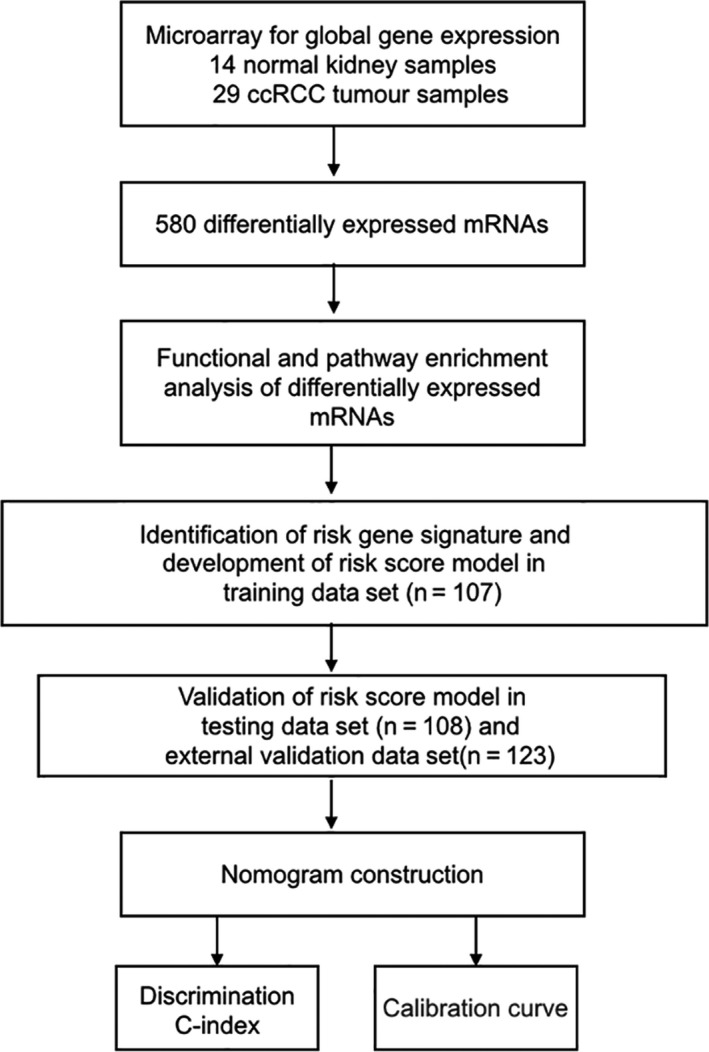
Study flow

We retrospectively analysed 215 paraffin‐embedded tumour tissues from ccRCC patients treated at the Sir Run Run Shaw Hospital (Hangzhou, China) between January 2004 and December 2008. Besides, we obtained a total of 123 ccRCC patients with global gene expression profiling and detailed clinical information from TCGA database serving as external validation data set (Table 1). Computer‐generated random numbers were applied to divide 215 specimens into a training cohort with a number of 107 samples and a validation cohort with a number of 108 samples. Total RNA was obtained from clinical FFPE samples by using the QIAGEN FFPE RNeasy kit (Qiagen GmbH). The quality of RNA was tested by NanoDrop 2000 spectrophotometer (ThermoFisher Scientific), and total RNA was amplified by Ovation FFPE WTA System (NuGEN). The specific study designs were shown in Figure 1. These studies were conducted with approval from the Ethics Committee.

**Table 1:** Patient characteristics of three cohorts

Variables	Training dataset (N = 107)	Testing dataset (N = 108)	External validation dataset (N = 123)
	N% or mean (range)		
Gender			
Male	73 (68.2)	62 (57.4)	68 (55.3)
Female	34 (31.8)	48 (42.6)	55 (44.7
Age (years)			
Male	58.16 (36‐79)	59.88 (37‐ 83)	57.98 (37‐79)
Female	60.61 (39‐82)	61.28 (33‐86)	60.56 (37‐82)
Tumor stage			
I	47 (43.9)	55 (50.9)	50 (40.7)
II	14 (13.1)	12 (11.1)	27 (22.0)
III	26 (24.3)	26 (24.1)	28 (22.8)
IV	20 (18.7)	15 (13.9)	18 (14.5
Fuhrman grade			
I	13 (12.1)	3 (2.8)	9 (7.2)
II	57 (53.2)	53 (49.1)	67 (54.6)
III	32 (29.9)	39 (36.1)	32 (26.0)
IV	5 (4.7)	13 (12.0)	15 (12.2)
Lymph node invasion	12 (11.2)	9 (8.3)	11 (8.9)
Necrosis	33 (30.8)	44 (40.7)	37 (30.1)
Number of events	48 (44.9)	38 (35.2)	58 (47.2)

### 2.2 Microarray data and differentially expressed gene analysis

ccRCC gene expression data (http://www.ncbi.nlm.nih.gov/geo/query/acc.cgi?acc=GSE68417) used in this study are available on GEO (https:// http://www.ncbi.nlm.nih.gov/geo/) 8. All raw data CEL files (Affymetrix Human Gene 1.0 ST Array) were processed under the same chip platform. These raw data files were downloaded and normalized by using a robust multi‐array averaging method (expresso(data,bgcorrect.method ="rma", normalize. method="quantiles", pmcorrect.method="pmonly", summary.method= "medianpolish")).^9^ A classical criterion of t test was adopted to identify DEGs with a change ≥twofold, and *P*‐value cut‐off <.01 was considered to be statistically significant.

### 2.3 Gene ontology analysis and Kyoto Encyclopedia of Genes and Genomes analysis

The Database for Annotation Visualization and Integrated Discovery (DAVID) was used to conduct the Gene ontology analysis (GO) and Kyoto Encyclopedia of Genes and Genomes (KEGG).^10^, ^11^ We used the human genome as the analysis background and defined *P* < .01 to be statistically significant.

### 2.4 Identification and validation of the prognostic gene signature

In order to screen out the risk gene signature, R software (version 3.2.1) and the ‘glmnet’ package were applied to perform the LASSO Cox regression analysis in the training data set. The LASSO penalty was used to achieve shrinkage and variable selection simultaneously, and the optimal values of the penalty parameter lambda were determined through 10 times cross‐validations. Genes which were significantly correlated with RFS in ccRCC were screen out based on the optimal lambda value. The risk score of each patient was calculated based on the expression level of each prognostic mRNA expression and its associated coefficient. Then, the patients in each data set were divided into low‐risk and high‐risk groups according to their mean risk score. Finally, we performed the Kaplan‐Meier estimator and the log‐rank test to assess RFS differences between above the low‐risk and high‐risk groups.

### 2.5 Validation of hub gene expression via quantitative real‐time PCR

The expression of identified hub genes was determined by qRT‐PCR. Fifty normal kidney samples and 50 ccRCC tumour samples were obtained from Sir Run Run Shaw Hospital for validation of hub genes solely. The mRNA expression levels of risk genes were normalized to an internal standard (glyceraldehyde‐3‐phosphate dehydrogenase, GAPDH). PCR primers used were as follows: DCN: forward, 5ʹ‐GACAAGGTCCGCCAGTTTATG‐3ʹ, reverse, 5ʹ‐TCGTCTAGTCTCCACTCATTCTG‐3ʹ; FGF2: forward, 5ʹ‐AGAAGAGCGACCCTCACATCA‐3ʹ, reverse, 5ʹ‐CGGTTAGCACACACTCCTTTG‐3ʹ; STAT6: forward, 5ʹ‐GTTCCGCCACTTGCCAATG‐3ʹ, reverse, 5ʹ‐TGGATCTCCCCTACTCGGTG‐3ʹ; CD19: forward, 5ʹ‐GGCCCGAGGAACCTCTAGT‐3ʹ, reverse, 5ʹ‐TAAGAAGGGTTTAAGCGGGGA‐3ʹ; MAP4K1: forward, 5ʹ‐GTCGTGGACCCTGACATTTTC‐3ʹ, reverse, 5ʹ‐CCTTAAAGACTTCCCCATACGTG‐3ʹ; GAPDH: forward, 5ʹ‐AGACAGCCGCATCTTCTTGT‐3ʹ, reverse, 5ʹ‐TGATGGCAACAATGTCCACT‐3ʹ.

### 2.6 Statistical analysis

We did a multivariate Cox regression analysis using backward selection to testify the independent of different indicators; variables (*P* < .05) were remained in the final model for nomogram construction.

Our nomogram was generated via rms package in R platform and the multivariable Cox regression model. Comparisons between the nomogram and other prognostic systems were performed by using the rcorrp.cens package in Hmisc in R. Statistical analysis was done in R (version 3.2.1) and SPSS (version 22.0). *P*‐value <.05 was deemed significant.

## 3 RESULTS

### 3.1 Identification of DEGs between normal kidney samples and ccRCC tumour samples

**Figure 2:**
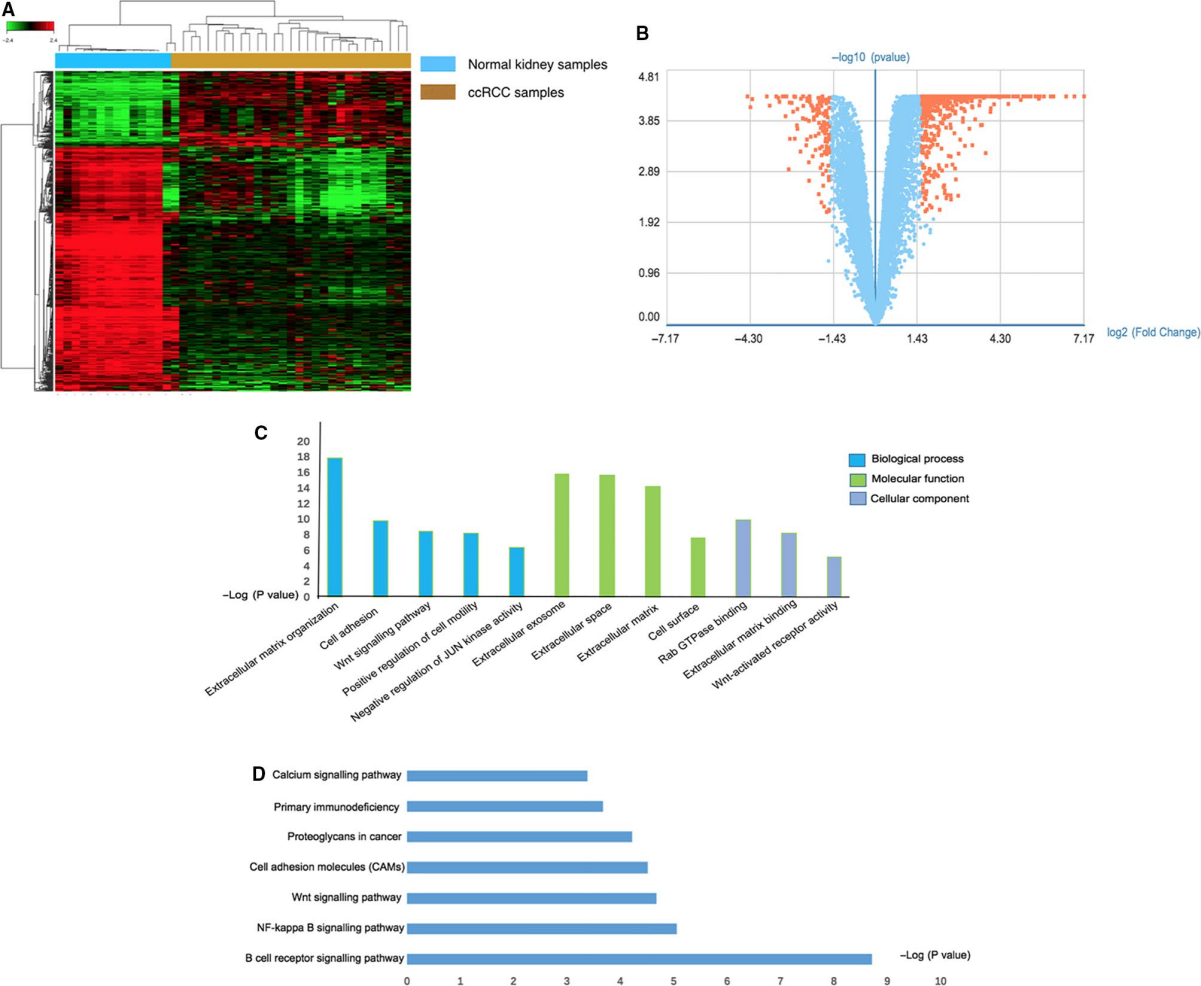
A, Heatmap of the differentially expressed genes. Clinical ccRCC samples versus normal kidney samples. Red: up‐regulation; green: down‐regulation. B, The volcano plot of the differentially expressed genes. C, Gene Ontology analysis and significant enriched GO terms related to ccRCC progression. D, Significantly enriched pathway terms related to progression

In the microarray analysis, with the criteria *P* < .01 and fold control (FC) ≥1.5, 580 genes were identified to be differentially expressed between 14 normal kidney samples and 29 ccRCC tumour samples. The volcano plot and heatmap were presented in Figure 2A and 2.

### 3.2 Functional enrichment analysis of DEGs and selection of risk gene signature

Then, 580 DEGs were put into DAVID for functional analysis. The GO analysis, including molecular function, cellular component (CC) and biological process (BP), showed that these genes were primarily involved in cell adhesion, positive regulation of cell motility and WNT signalling pathway (Figure 2C). To further elucidate the potential functional pathways of DEGs, we conducted KEGG pathway enrichment analysis. B cell receptor signalling pathway, NF‐kappa B signalling pathway and WNT signalling pathway were considered to be the most significantly enriched pathways (Figure 2D). LASSO Cox regression was used for further analysis, and 5 of these differentially expressed genes were identified to be significantly related to RFS of ccRCC (Table 2). The risk scores of each patient were calculated by a formula which was derived from the expression level of five risk genes weighted by regression coefficient.

**Table 2:** mRNA significantly associated with the recurrence‐free survival in Training dataset. T/N: expression in ccRCC samples/expression in normal kidney samples

Variables	Expression(T/N)	HR	*P* value	Coefficient	Description
STAT6	Up‐regulated	4.014	<.0001	1.674	Signal transducer and activator of transcription 6
CD19	Up‐regulated	1.817	<.0001	0.749	CD19 molecule
MAP4K1	Up‐regulated	1.802	.004	0.714	Mitogen‐activated protein kinase kinase kinase kinase 1
FGF2	Down‐regulated	1.674	.005	−0.643	Fibroblast growth factor 2
DCN	Down‐regulated	1.798	.007	−0.756	Decorin

### 3.3 To further demonstrate the expression of risk genes via RT‐QPCR

**Figure 3:**
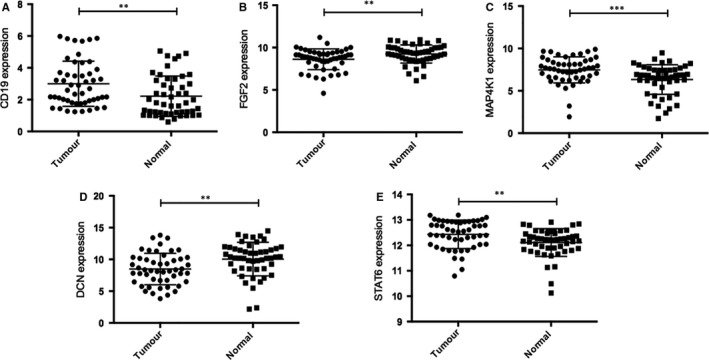
To further confirm the expression of identified hub genes in clinical samples: CD19, FGF2, MAP4K1, DCN and STAT6

To further confirm the expression of identified risk genes from network‐based analysis, RT‐QPCR assay of five hub genes (CD19, FGF2, MAP4K1, DCN and STAT6) were performed between 50 normal kidney samples and 50 ccRCC tumour samples. The mRNA expression levels of five hub genes were consistent with microarray results (Figure 3). In conclusion, these results indicated that these five risk genes (CD19, FGF2, MAP4K1, DCN and STAT6) were actually differentially expressed between normal kidney samples and ccRCC tumour samples.

### 3.4 Construction and validation of risk gene signature score model for predicting RFS of ccRCC patients

**Figure 4:**
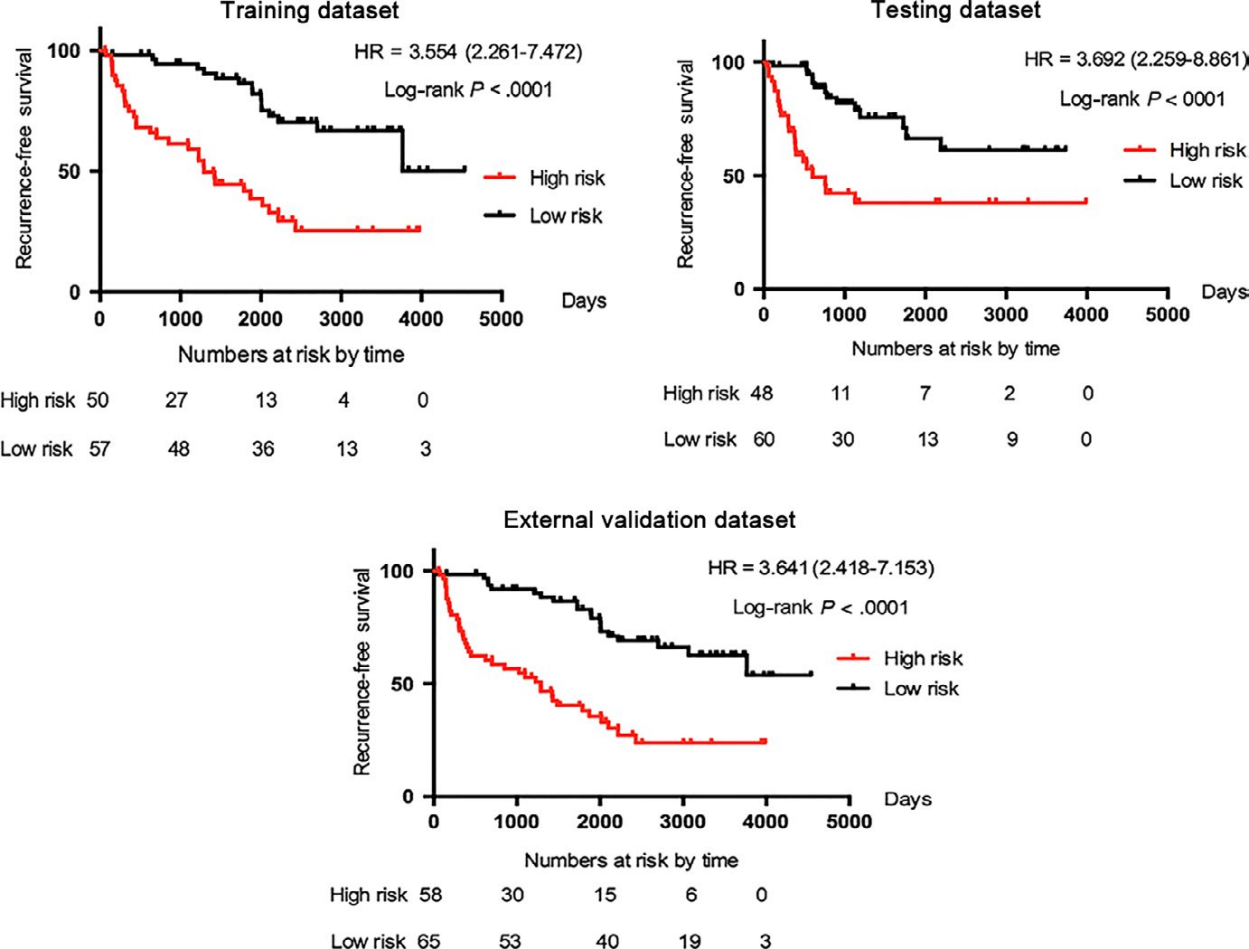
Kaplan‐Meier curves of recurrence‐free survival according to the risk gene signature. A, Training cohort (n = 107); B, testing cohort (n = 108); C, external validation cohort (n = 123). *P* values and hazard ratios were calculated by using the unadjusted log‐rank test. HR = hazard ratio

The 215 patients were randomly divided into training data set (n = 107) and testing data set (n = 108). Median follow‐up was 61.4 months (IQR 87.7‐23.1) for patients in the training data set and 37.1 months (IQR 51.7‐25.2) for those in the testing data set. The patients in training data set were divided into low‐risk group (n = 57) and high‐risk group (n = 50) based on the mean risk score. Patients in the high‐risk group indicated a worse clinical prognosis when compared with those in the low‐risk group (Figure 4A). The efficacy of our five gene‐signature for RFS prediction of ccRCC patients was further verified in testing data set and external validation data set. The same risk gene score‐based classifier was used to classify patients in testing data set and external validation data set into the high‐risk and the low‐risk groups. Consistent with the results described above, patients in the high‐risk group had a significantly shorter RFS (Figure 4B and 4).

### 3.5 Independence of 5‐gene signature risk score model for RFS prediction from clinical features

To determine whether the prognostic value of five gene‐signature was independent of patient clinical features, we performed the univariable and multivariate Cox regression analyses using RFS as the dependent variable and five gene‐signature score, age, gender, tumour stage, grade, lymph node invasion and necrosis as covariates in each data set. We found that the five gene‐signature was significantly related to RFS of ccRCC patients after adjusting for clinical features in the training data set (HR = 2.107, CI = 1.689‐2.773, *P* = .002), the testing data set (HR = 2.418, CI = 1.683‐3.417, *P* < .0001) and the external validation data set (HR = 2.195, CI = 1.645‐2.595, *P* < .0001) (Tables 3, 4, 5).

**Table 3:** Univariable and multivariable Cox regression analysis of risk gene signature and other clinical features in training dataset

Variables	Univariate analysis			Multivariate analysis		
	HR	95% CI of HR	*P* value	HR	95% CI of HR	*P* value
risk gene signature	2.509	1.990‐3.163	<.0001	2.107	1.689‐2.773	.002
Age	1.003	0.976‐1.030	.834	0.993	0.965‐1.021	.615
Gender	1.023	0.572‐1.831	.938	1.065	0.559‐2.028	.849
Tumour stage	2.475	1.826‐3.354	<.0001	1.490	1.056‐2.007	.019
Fuhrman grade	3.091	1.979‐4.826	<.0001	1.713	1.016‐2.887	.031
Necrosis	5.857	3.159‐10.859	<.0001	2.075	1.012‐4.256	.043
Lymph node invasion	16.233	7.369‐35.761	<.0001	2.972	1.176‐7.510	.021

**Table 4:** Univariable and multivariable Cox regression analyses of risk gene signature and other clinical features in testing dataset

Variables	Univariate analysis			Multivariate analysis		
	HR	95% CI of HR	*P* value	HR	95% CI of HR	*P* value
risk gene signature	2.727	2.011‐3.914	<.0001	2.418	1.683‐3.417	<.0001
Age	1.005	0.979‐1.032	.728	1.026	0.995‐1.059	.105
Gender	1.008	0.514‐1.975	.981	0.782	0.352‐1.740	.782
Tumour stage	2.262	1.795‐3.524	<.0001	2.228	1.442‐3.442	<.0001
Fuhrman grade	2.642	1.732‐4.031	<.0001	2.153	1.245‐3.724	.006
Necrosis	4.561	2.214‐9.397	<.0001	2.326	1.050‐5.154	.038
Lymph node invasion	5.327	2.403‐11.809	<.0001	3.495	1.400‐8.723	.007

**Table 5:** Univariable and multivariable Cox regression analyses of risk gene signature and other clinical features in external validation dataset

Variables	Univariate analysis			Multivariate analysis		
	HR	95% CI of HR	*P* value	HR	95% CI of HR	*P* value
risk gene signature	2.922	2.570‐3.354	<.0001	2.195	1.645‐2.595	<.0001
Age	1.007	0.983‐1.032	.570	0.999	0.974‐1.024	.935
Gender	1.363	0.801‐2.320	.254	1.378	0.788‐2.410	.261
Tumour stage	3.142	2.335‐4.226	<.0001	1.519	1.032‐2.236	.034
Fuhrman grade	4.354	2.873‐6.598	<.0001	2.245	1.239‐3.879	.004
Necrosis	4.251	2.474‐7.302	<.0001	2.545	1.395‐4.644	.002
Lymph node invasion	8.721	4.132‐18.409	<.0001	2.791	1.253‐6.219	.012

**Figure 5:**
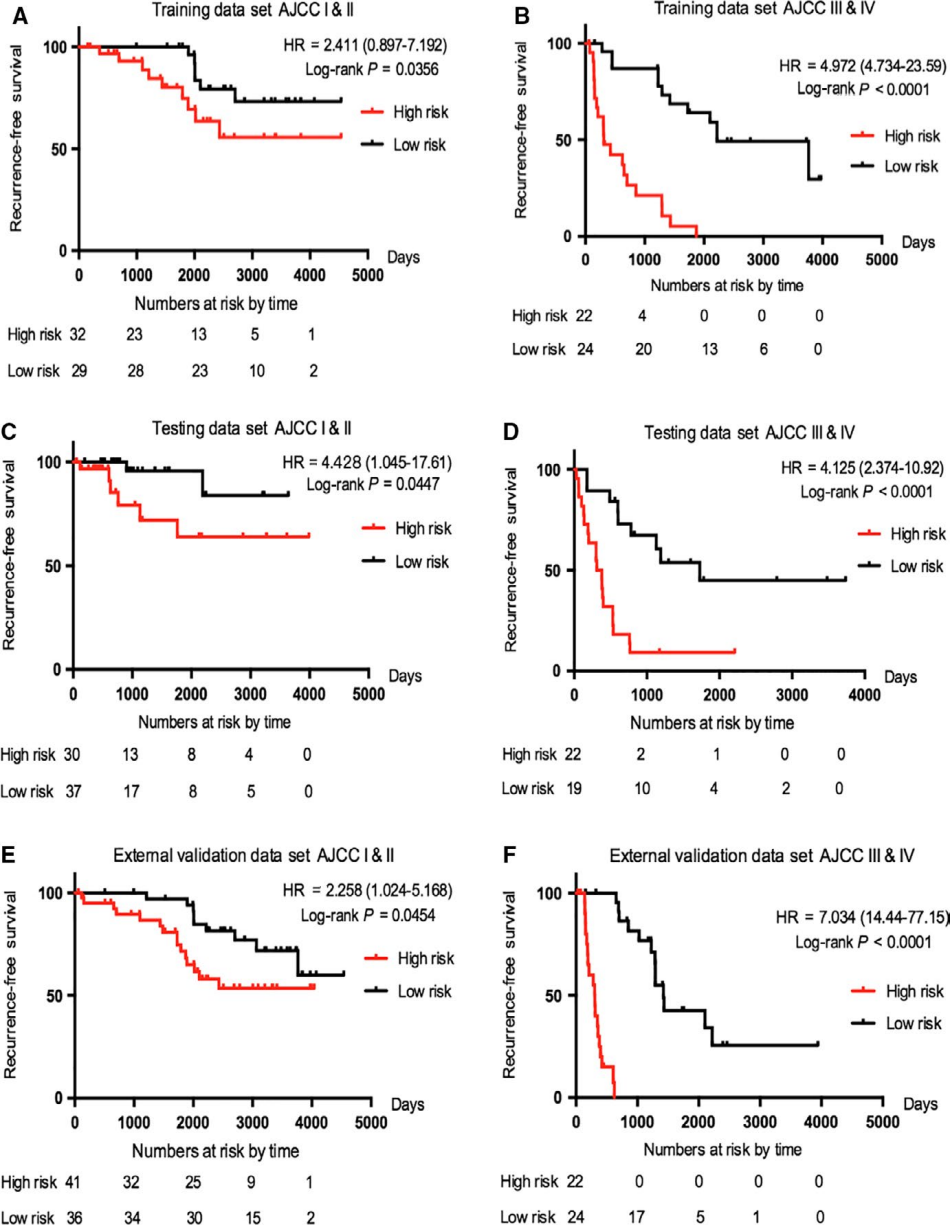
Using Kaplan‐Meier survival analysis to testify the independence of our risk gene signature from AJCC stage. The patients from each data set were stratified into subgroups. The risk gene signature was applied to the low‐stage patients (A, C, E) and high‐stage patients (B, D, F)

Besides, we introduced the stratification based on tumour stage. We further stratified ccRCC patients into two subgroups where the AJCC stages I and II were fictitiously described as an early‐stage stratum and the AJCC stages III and IV as a late‐stage stratum. Result from Figure 5A‐F indicated that the risk gene signature still had the ability to distinguish that the outcome of patients with high‐risk score was dramatically worse than that with low‐risk score both in the early‐stage and late‐stage stratums.

**Figure 6:**
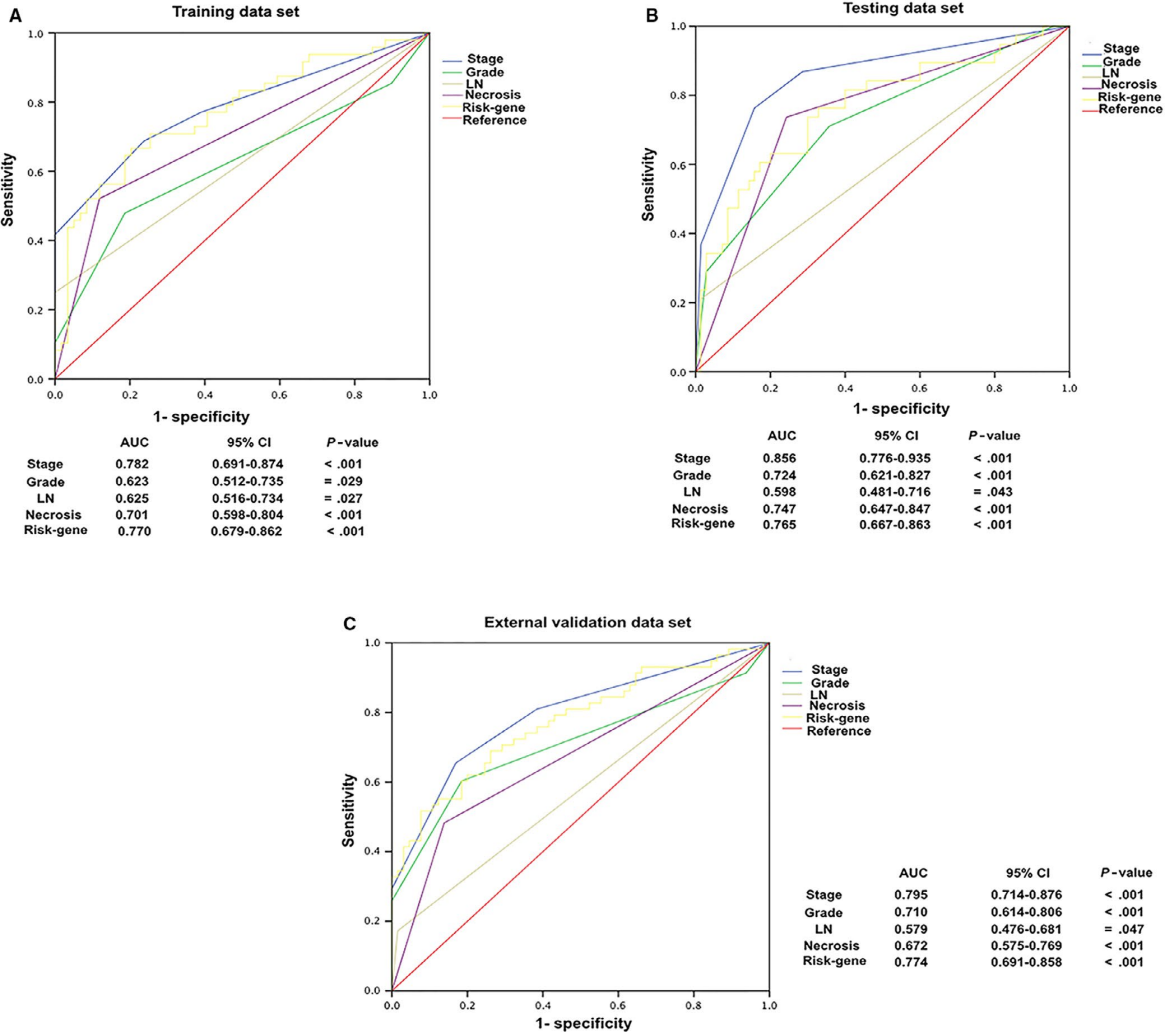
Receiver operating characteristic (ROC) analysis was performed to evaluate the specificity and sensitivity of the RFS prediction by risk gene score, tumour stage, tumour grade, lymph node invasion and necrosis in (A) training cohort (n = 107); (B) testing cohort (n = 108) and (C) external validation cohort (n = 123)

Receiver operating characteristic analysis was also performed to testify the specificity and sensitivity of RFS prediction in each data set (Figure 6A‐C). The risk gene signature score model possessed a similar predictive power compared with AJCC stage and tumour grade for the prognostic evaluation of ccRCC patients.

### 3.6 Construction of nomogram combined 5‐gene signature with the other clinical features for personalized prediction

**Figure 7:**
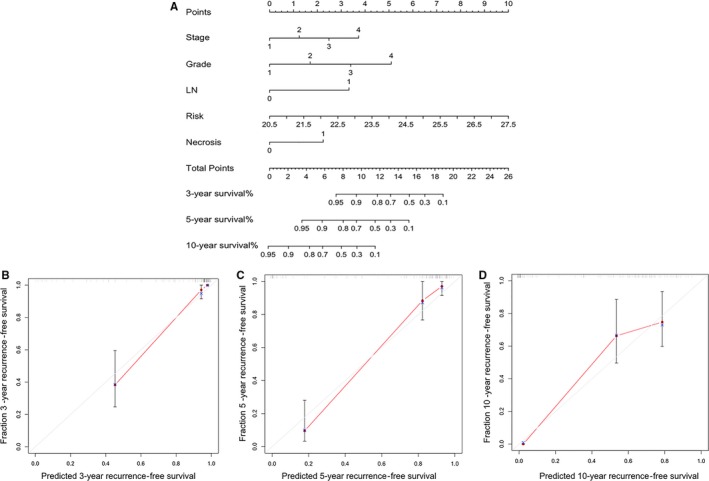
The nomogram for personalized prediction of RFS in ccRCC patients. (A) The nomogram for predicting probability of patients with recurrence‐free survival. Risk: Risk gene signature scores; LN: lymph node invasion (B) The calibration plots for predicting RFS at 3 years. The calibration plots for predicting RFS at 5 years (C) and 10 years (D). Nomogram‐predicted probability of recurrence is plotted on the *x*‐axis; actual recurrence is plotted on the y‐axis. The red line represents the predictive efficacy of our nomogram

To come up with a useful approach to predict the risk of recurrence so as to facilitate personalized management of ccRCC patients, we constructed a nomogram which combined our five gene‐signature, and clinical features for predicting 3‐year, 5‐year and 10‐year RFS (Figure 7A). Results from Figure 7B‐D indicated that the line segments in the calibration plots were close to the 45° line which meant the well prediction, demonstrating that our nomogram was useful for prediction of 3‐year, 5‐year and 10‐year RFS. Besides, the C‐index of our nomogram was 0.859, which also indicated the high predictive accuracy.

Our nomogram combined both genomic and clinical features of ccRCC patients, which showed better accuracy for predicting RFS of ccRCC after nephrectomy when compared with SSIGN prognostic system.^12^ The C‐index of our nomogram was 0.859, 95% confidence interval (CI) 0.8149‐0.9031, which was significantly higher (*P* < .01) than the SSIGN prognostic system (0.808), 95% CI 0.758‐0.858. Besides, while we excluded the risk gene signature from the nomogram, the C‐index of nomogram dropped to 0.819, 95% CI 0.775‐0.863. These results indicated that our nomogram could serve as a predictor for RFS of ccRCC.

## 4 DISCUSSION

In this retrospective study, results indicated that our risk gene signature model developed in this research could categorize patients who had significantly different RFS into the low‐ and high‐risk groups. Besides, the efficacy of risk gene signature was verified in both internal and external validation cohorts, respectively. Importantly, a novel prognostic nomogram for precisely predicting RFS of ccRCC patients after nephrectomy was constructed based on the expression of risk gene signature and clinical risk features.

Cancers are recognized as heterogeneous disease.^13^ Thus, identifying the dysregulated genes in tumour carcinogenesis and progression could be helpful for improving prognostic and therapeutic strategies.^14^ Nowadays, development in microarray has contribute to the acquisition of large amounts of data which is useful for exploring molecular mechanisms, risk stratification and guiding strategies for clinical therapy in different cancers.^15^, ^16^, ^17^ In our study, microarray analysis was performed to acquire different expressed genes between normal kidney and ccRCC. Risk gene signature classifier was generated to predict recurrence risk, and its prognostic value was verified in both internal and external validation cohorts. Moreover, this indicator is independent of clinical features and possessed a similar predictive power compared with those widely used indicators for ccRCC such as AJCC stage and tumour grade. Among these genes, MAP4K1 was previously known to positively regulate cell motility and thereby to influence tumour cell invasion in the medulloblastoma and colon carcinoma.^18^, ^19^, ^20^ Beside, Lourdes and Wang, Y indicated that the MAP4K1 was related to the progression of bladder cancer^21^, ^22^; STAT6 was found to promote intestinal tumorigenesis in the mouse model via inhibition of cytotoxic CD8 response^23^ and was involved in lymphoma.^24^, ^25^ It is reported that activation of FGFR1 by its ligand fibroblast growth factor 2 (FGF2) could promote cell proliferation, epithelial‐mesenchymal transition and invasion in lung cancer.^26^ Overexpression of FGF2 could also induce EMT in malignant pleural mesothelioma cells via MAPK/MMP1 signal and confer the poor prognosis.^27^ DCN is found to be a novel biomarker for the diagnosis of colon cancer by using iTRAQ‐tagging and 2D‐LC‐MS/MS.^28^ Researchers found that T cells with chimeric antigen receptors (CAR T cells) which targets human CD19 (hCD19) have shown great efficacy against B cell malignancies.^29^ Therefore, our risk gene signature could potentially serve as a predictive appliance for personalized treatment and might also be potential target for clinical therapeutic targets of ccRCC.

Finally, a nomogram was constructed for prediction of an individual's recurrence risk. Despite the fact that we, nowadays, often apply traditional indicators in clinic, such as tumour grade, stage and lymph node invasion (lymph node invasion owns highest hazard ratios in our study), these factors are unable to guide personalized treatment.^30^, ^31^ Importantly, when patients presented with the same stage or grade, these traditional factors are unable to predict an individual's risk. Therefore, our nomogram combined individual gene signature reflecting the biological heterogeneity of different ccRCC patients with traditional prognostic factors providing insights into a patient's clinicopathologic features so as to elevated the accuracy of individual RFS prediction.^32^, ^33^ We also demonstrated the performance of our nomogram in validation cohorts. However, this research is retrospective and our sample size is still limited. Thus, our risk gene model and nomogram still require further validation in multicenter clinical trials. Besides, we will validate the efficiency of our nomogram in other ccRCC patient cohorts in the following studies.

## 5 CONCLUSIONS

This is the first research to combine gene expression profiles with clinical information for predicting clinical prognosis of ccRCC patients. Our results show that the risk gene signature can effectively classify ccRCC patients into high and low‐risk groups. Moreover, this nomogram might help clinicians accurately and personally predict the prognosis of patients with ccRCC after nephrectomy.

## CONFLICT OF INTEREST

The authors declare no conflict of interest.

## AUTHOR CONTRIBUTIONS

YLC, SJJ, LQX, JYL and GHL conceptualized the data; WH, YLC and SJJ contributed to methodology; LQX, LYL and DWX provided software; YLC, SJJ and ZYL investigated the study; LQX and LYL provided resources; YLC and SJJ curated the data; YLC wrote—original draft preparation; YLC wrote—review and editing; ZYL and LWX visualized the data; GHL supervised the study; GHL administrated the project; GHL acquired funding.

## Data Availability

The data that support the findings of this study are openly available in GEO (https:// http://www.ncbi.nlm.nih.gov/geo/) and TCGA (https://www.cancer.gov/about-nci/organization/ccg/research/structural-genomics/tcga).

## References

[1] Siegel R , Naishadham D , Jemal A . Cancer statistics, 2013. CA Cancer J Clin. 2013;63:11‐30. 23335087 10.3322/caac.21166

[2] Comperat E , Camparo P . Histological classification of malignant renal tumours at a time of major diagnostic and therapeutic changes. Diagn Interv Imaging. 2012;93:221‐231. 22465787 10.1016/j.diii.2012.01.015

[3] Li B , Qiu B , Lee DS , et al. Fructose‐1,6‐bisphosphatase opposes renal carcinoma progression. Nature. 2014;513:251‐255. 25043030 10.1038/nature13557 PMC4162811

[4] Ficarra V , Galfano A , Mancini M , Martignoni G , Artibani W . TNM staging system for renal‐cell carcinoma: current status and future perspectives. Lancet Oncol. 2007;8:554‐558. 17540307 10.1016/S1470-2045(07)70173-0

[5] Ramaswamy V , Remke M , Bouffet E , et al. Recurrence patterns across medulloblastoma subgroups: an integrated clinical and molecular analysis. Lancet Oncol. 2013;14:1200‐1207. 24140199 10.1016/S1470-2045(13)70449-2 PMC3953419

[6] Brooks SA , Khandani AH , Fielding JR , et al. Alternate metabolic programs define regional variation of relevant biological features in renal cell carcinoma progression. Clin Cancer Res. 2016;22:2950‐2959. 26787754 10.1158/1078-0432.CCR-15-2115 PMC4911278

[7] Verbiest A , Couchy G , Job S , et al. Molecular subtypes of clear‐cell renal cell carcinoma are prognostic for outcome after complete metastasectomy. Eur Urol. 2018;74(4):474‐480. 10.1016/j.eururo.2018.01.042. 29463434

[8] Barrett T , Troup DB , Wilhite SE , et al. NCBI GEO: archive for high‐throughput functional genomic data. Nucleic Acids Res. 2009;37:D885‐D890. 18940857 10.1093/nar/gkn764 PMC2686538

[9] Irizarry RA , Hobbs B , Collin F , et al. Exploration, normalization, and summaries of high density oligonucleotide array probe level data. Biostatistics. 2003;4:249‐264. 12925520 10.1093/biostatistics/4.2.249

[10] Sherman BT , da Huang W , Tan Q , et al. DAVID Knowledgebase: a gene‐centered database integrating heterogeneous gene annotation resources to facilitate high‐throughput gene functional analysis. BMC Bioinformatics. 2007;8:426. 17980028 10.1186/1471-2105-8-426 PMC2186358

[11] Kanehisa M , Goto S . KEGG: Kyoto encyclopedia of genes and genomes. Nucleic Acids Res. 2000;28(1):27‐30. 10592173 10.1093/nar/28.1.27 PMC102409

[12] Zigeuner R , Hutterer G , Chromecki T , et al. External Validation of the Mayo Clinic Stage, Size, Grade, and Necrosis (SSIGN) Score for Clear‐Cell Renal Cell Carcinoma in a Single European Centre Applying Routine Pathology. Eur Urol. 2010;57:102‐111. 19062157 10.1016/j.eururo.2008.11.033

[13] Rajpert‐De ME . Developmental model for the pathogenesis of testicular carcinoma in situ: genetic and environmental aspects. Hum Reprod Update. 2006;12:303‐323. 16540528 10.1093/humupd/dmk006

[14] Sestak I , Cuzick J , Dowsett M , et al. Prediction of late distant recurrence after 5 years of endocrine treatment: a combined analysis of patients from the Austrian breast and colorectal cancer study group 8 and arimidex, tamoxifen alone or in combination randomized trials using the PAM50 risk of recurrence score. J Clin Oncol. 2015;33:916‐922. 25332252 10.1200/JCO.2014.55.6894

[15] Tsai M , Lo S , Audeh W , et al. Association of 70‐gene signature assay findings with physicians' treatment guidance for patients with early breast cancer classified as intermediate risk by the 21‐gene assay. JAMA Oncol. 2018;4:e173470. 29075751 10.1001/jamaoncol.2017.3470 PMC5833645

[16] Zhao SG , Chang SL , Spratt DE , et al. Development and validation of a 24‐gene predictor of response to postoperative radiotherapy in prostate cancer: a matched, retrospective analysis. Lancet Oncol. 2016;17:1612‐1620. 27743920 10.1016/S1470-2045(16)30491-0

[17] Rini B , Goddard A , Knezevic D , et al. A 16‐gene assay to predict recurrence after surgery in localised renal cell carcinoma: development and validation studies. Lancet Oncol. 2015;16:676‐685. 25979595 10.1016/S1470-2045(15)70167-1

[18] Grunder E , D'Ambrosio R , Fiaschetti G , et al. MicroRNA‐21 suppression impedes medulloblastoma cell migration. Eur J Cancer. 2011;47:2479‐2490. 21775132 10.1016/j.ejca.2011.06.041

[19] Yang HS , Matthews CP , Clair T , et al. Tumorigenesis suppressor Pdcd4 down‐regulates mitogen‐activated protein kinase kinase kinase kinase 1 expression to suppress colon carcinoma cell invasion. Mol Cell Biol. 2006;26:1297‐1306. 16449643 10.1128/MCB.26.4.1297-1306.2006 PMC1367180

[20] Meng Z , Moroishi T , Mottier‐Pavie V , et al. MAP4K family kinases act in parallel to MST1/2 to activate LATS1/2 in the Hippo pathway. Nat Commun. 2015;6:8357. 26437443 10.1038/ncomms9357 PMC4600732

[21] van der Heijden AG , Mengual L , Lozano JJ , et al. A five‐gene expression signature to predict progression in T1G3 bladder cancer. Eur J Cancer. 2016;64:127‐136. 27414486 10.1016/j.ejca.2016.06.003

[22] Wang Y , Luo H , Li Y , Chen T , Wu S , Yang L . hsa‐miR‐96 up‐regulates MAP4K1 and IRS1 and may function as a promising diagnostic marker in human bladder urothelial carcinomas. Mol Med Rep. 2012;5:260‐265. 21993544 10.3892/mmr.2011.621

[23] Jayakumar A , Bothwell A . Stat6 promotes intestinal tumorigenesis in a mouse model of adenomatous polyposis by expansion of MDSCs and inhibition of cytotoxic CD8 response. Neoplasia. 2017;19:595‐605. 28654863 10.1016/j.neo.2017.04.006 PMC5487300

[24] Spina V , Bruscaggin A , Cuccaro A , et al. Circulating tumor DNA reveals genetics, clonal evolution and residual disease in classical Hodgkin lymphoma. Blood. 2018;131(22):2413‐2425. 10.1182/blood-2017-11-812073 29449275

[25] Scott LM , Gandhi MK . Deregulated JAK/STAT signalling in lymphomagenesis, and its implications for the development of new targeted therapies. Blood Rev. 2015;29:405‐415. 26123794 10.1016/j.blre.2015.06.002

[26] Wang K , Ji W , Yu Y , et al. FGFR1‐ERK1/2‐SOX2 axis promotes cell proliferation, epithelial‐mesenchymal transition, and metastasis in FGFR1‐amplified lung cancer. Oncogene. 2018;37:5340‐5354. 29858603 10.1038/s41388-018-0311-3

[27] Schelch K , Wagner C , Hager S , et al. FGF2 and EGF induce epithelial–mesenchymal transition in malignant pleural mesothelioma cells via a MAPKinase/MMP1 signal. Carcinogenesis. 2018;39(4):534‐545. 10.1093/carcin/bgy018 29635378

[28] Li G , Li M , Liang X , et al. Identifying DCN and HSPD1 as potential biomarkers in colon cancer using 2D‐LC‐MS/MS combined with iTRAQ technology. J Cancer. 2017;8:479‐489. 28261350 10.7150/jca.17192 PMC5332900

[29] Turtle CJ , Hay KA , Hanafi LA , et al. Durable molecular remissions in chronic lymphocytic leukemia treated with CD19‐specific chimeric antigen receptor‐modified T cells after failure of ibrutinib. J Clin Oncol. 2017;35:3010‐3020. 28715249 10.1200/JCO.2017.72.8519 PMC5590803

[30] Huitzil‐Melendez FD , Capanu M , O'Reilly EM , et al. Advanced hepatocellular carcinoma: which staging systems best predict prognosis? J Clin Oncol. 2010;28:2889‐2895. 20458042 10.1200/JCO.2009.25.9895 PMC3651603

[31] Fouad TM , Barrera A , Reuben JM , et al. Inflammatory breast cancer: a proposed conceptual shift in the UICC‐AJCC TNM staging system. Lancet Oncol. 2017;18:e228‐e232. 28368261 10.1016/S1470-2045(17)30192-4 PMC6140765

[32] Wang Y , Li J , Xia Y , et al. Prognostic nomogram for intrahepatic cholangiocarcinoma after partial hepatectomy. J Clin Oncol. 2013;31:1188‐1195. 23358969 10.1200/JCO.2012.41.5984

[33] Wierda WG , O'Brien S , Wang X , et al. Prognostic nomogram and index for overall survival in previously untreated patients with chronic lymphocytic leukemia. Blood. 2007;109:4679‐4685. 17299097 10.1182/blood-2005-12-051458

